# Evasion of regulatory phosphorylation by an alternatively spliced isoform of Musashi2

**DOI:** 10.1038/s41598-017-11917-3

**Published:** 2017-09-14

**Authors:** Melanie C. MacNicol, Chad E. Cragle, F. Kennedy McDaniel, Linda L. Hardy, Yan Wang, Karthik Arumugam, Yasir Rahmatallah, Galina V. Glazko, Ania Wilczynska, Gwen V. Childs, Daohong Zhou, Angus M. MacNicol

**Affiliations:** 1 University of Arkansas for Medical Sciences, Department of Neurobiology and Developmental Sciences, 4301 W. Markham, Little Rock, 72205 AR USA; 2 University of Arkansas for Medical Science, Center for Translational Neuroscience, 4301 W. Markham, Little Rock, 72205 AR USA; 3 University of Arkansas for Medical Sciences, Department of Pharmaceutical Sciences, 4301 W. Markham, Little Rock, 72205 AR USA; 4 University of Arkansas for Medical Sciences, Department of Physiology and Biophysics, 4301 W. Markham, Little Rock, 72205 AR USA; 5 University of Arkansas for Medical Sciences, Department of Biomedical Informatics, 4301 W. Markham, Little Rock, 72205 AR USA; 6 MRC Toxicology Unit, Lancaster Road, Leicester, UK; 7 Winthrop P. Rockefeller Cancer Institute, University of Arkansas for Medical Sciences, 4301 W. Markham, Little Rock, AR 72205 United States; 8 Center for Genomic Regulation, Department of Gene Regulation, Stem Cells and Cancer, C/Dr. Aiguader 88, 08003 Barcelona, Spain; 9 Present Address: Department of Orthopedics, Guangzhou First People’s Hospital, Guangzhou Medical University, Guangzhou, Guangdong 510182 PR China

## Abstract

The Musashi family of RNA binding proteins act to promote stem cell self-renewal and oppose cell differentiation predominantly through translational repression of mRNAs encoding pro-differentiation factors and inhibitors of cell cycle progression. During tissue development and repair however, Musashi repressor function must be dynamically regulated to allow cell cycle exit and differentiation. The mechanism by which Musashi repressor function is attenuated has not been fully established. Our prior work indicated that the Musashi1 isoform undergoes site-specific regulatory phosphorylation. Here, we demonstrate that the canonical Musashi2 isoform is subject to similar regulated site-specific phosphorylation, converting Musashi2 from a repressor to an activator of target mRNA translation. We have also characterized a novel alternatively spliced, truncated isoform of human Musashi2 (variant 2) that lacks the sites of regulatory phosphorylation and fails to promote translation of target mRNAs. Consistent with a role in opposing cell cycle exit and differentiation, upregulation of Musashi2 variant 2 was observed in a number of cancers and overexpression of the Musashi2 variant 2 isoform promoted cell transformation. These findings indicate that alternately spliced isoforms of the Musashi protein family possess distinct functional and regulatory properties and suggest that differential expression of Musashi isoforms may influence cell fate decisions.

## Introduction

Targeted control of mRNA translation is gaining recognition as a key mechanism for regulation of cell cycle and cell fate transitions^1–5^. This form of regulation of gene expression permits a rapid cellular response to changing external cues through repression or translation of specific pre-existing mRNAs. Target mRNA specificity is achieved through sequence-specific targeting of RNA binding proteins (RBPs) and/or miRNAs that modulate the stability and/or translation of the target mRNA. The mechanisms by which the function of RBPs are regulated are not well understood but are of increasing interest, as it has become evident that aberrant control of mRNA translation contributes to a range of pathologies, including neurological disease and cancer^6–10^.

The two Musashi (Msi) RBP protein family members, Musashi1 (Msi1) and Musashi2 (Msi2), have been identified as mediators of both physiological and pathological stem cell self-renewal^11–23^. Msi is thought to promote stem cell self-renewal and opposes cell cycle arrest and cell differentiation by repressing the translation of key target mRNAs^12^. Identified mammalian targets of Msi-mediated repression include the mRNAs encoding Numb, a Notch signaling inhibitor; p21, an inhibitor of cyclin-dependent kinases; adenomatous polypopsis coli, a Wnt signaling inhibitor; doublecortin, a protein associated with neuronal migration and development; and Dnmt1, a DNA methylating enzyme responsible for maintenance of epigenetic marks^24–28^. The mechanism by which Msi target mRNAs are de-repressed during developmental processes or tissue repair to allow cell cycle exit and stem/progenitor cell differentiation is not fully understood^29^. The low level of Msi proteins in terminally differentiated, mature cells suggests that target de-repression could be mediated through simple degradation of Msi protein. However, it has been observed that de-repression of Msi target mRNAs precedes loss of Msi protein, suggesting that alternate mechanisms act to regulate Msi function^24, 29, 30^. Moreover, there is evidence that the Msi1 isoform can switch function to activate, rather than repress, translation of target mRNAs. Target mRNA activation was first shown in oocytes of the frog, *Xenopus*, where pro-differentiation signaling triggered Msi1-dependent translation of target mRNAs including Mos, a MAP kinase signaling activator; cyclin B5, Msi1 itself and Dnmt1^28, 31–33^. While subsequent studies have reported specific examples of Msi1- and Msi2-dependent translation of mammalian target mRNAs^34–39^, it is unclear the extent to which Msi1 and Msi2 are controlled by similar regulatory mechanisms during cell fate transitions.

The Msi1 and Msi2 proteins each contain two highly conserved N-terminal RNA recognition motifs (RRMs), and recent crosslinking studies indicate that they may regulate overlapping mRNA target cohorts^40^. Consistent with possible shared mRNA targets, Msi1 and Msi2 have been shown to effectively substitute for each other in the control of *Xenopus* oocyte maturation, mammalian neuronal stem cell self-renewal, intestinal stem cell quiescence and colorectal cancer^32, 40–42^. Despite these apparent similarities, several lines of evidence suggest differences between the Msi family members, in terms of expression patterns, as well as interaction with protein binding partners and function. While co-expressed in many tissues, Msi2 is selectively expressed in hematopoietic stem cells^43^. Mammalian Msi2 does not appear to interact with the Msi1-associated proteins Lin28 or GLD2 poly[A] polymerase^44, 45^ and it has been reported that Msi2 opposes proliferation in pancreatic cells while Msi1 acts to promote proliferation^46^. Together, these observations suggest that Msi1 and Msi2 may be subject to both shared as well as isoform-specific regulatory mechanisms.

In this study, we characterized the regulatory control of the Msi2 protein. We report that Msi2 undergoes stimulus-dependent phosphorylation on two conserved serine residues during maturation of *Xenopus* oocytes, as well as during differentiation of mammalian cells in culture. We demonstrate that Msi2 phosphorylation is mediated by both Ringo/CDK signaling and p42 MAP kinase (ERK) signaling pathways and that mutational disruption of Msi2 phosphorylation abrogates stimulus-dependent target mRNA translational activation and *Xenopus* oocyte maturation. Msi2 phosphorylation results in the translational activation of previously characterized Msi1 target mRNAs. Importantly, we characterized an alternatively spliced, truncated isoform of human Msi2 (variant 2) that lacks the exon containing the two conserved sites of regulatory phosphorylation. Expression of Msi2 variant 2 fails to promote target mRNA translation and is non-permissive for maturation of *Xenopus* oocytes. Overexpression of the Msi2 variant 2 promotes growth and cellular transformation of NIH3T3 cells, consistent with the Msi2 variant 2 failing to promote de-repression and translation of target mRNAs that inhibit cell proliferation. Our results indicate that the canonical Msi1 and Msi2 proteins have shared regulatory mechanisms while highlighting an unexpected role for alternative splicing in Msi2 isoform-specific control.

## Results

### Regulated phosphorylation controls *Xenopus* Msi2 function

**Figure 1:**
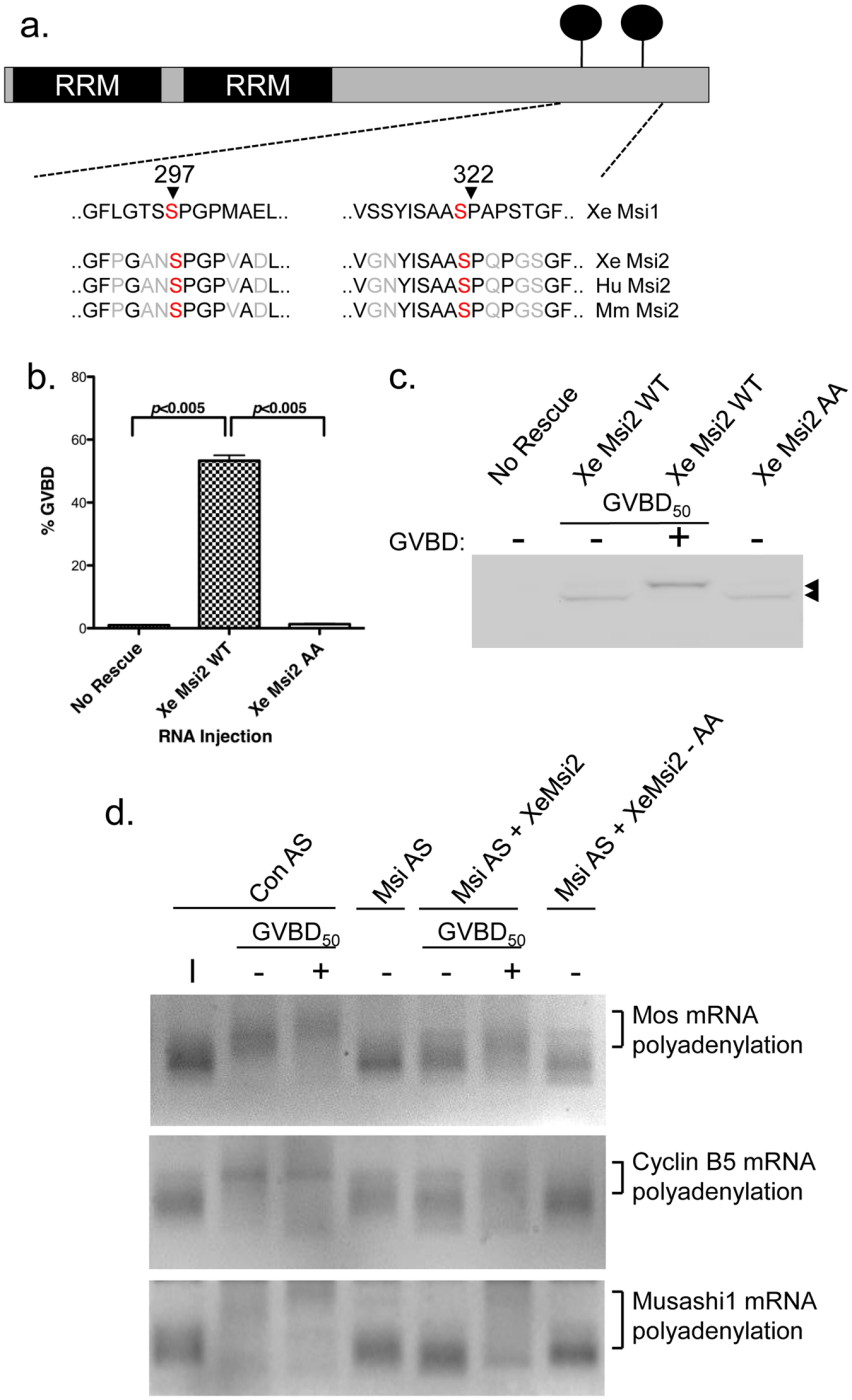
Regulatory phosphorylation of Msi2 controls translational activation of target mRNAs and oocyte maturation. (**a**) Schematic alignment of *Xenopus* Msi1 regulatory phosphorylation motifs with similar motifs in Msi2 isoforms from *Xenopus* (Xe), Human (Hu) and mouse (Mm). (**b**) Immature stage VI oocytes were injected with antisense oligonucleotides to Msi1 and Msi2, incubated overnight and subsequently re-injected with water (No Rescue), or RNA encoding *Xenopus* Msi2 wild type (Msi2 WT) or mutant Msi2 S356A/S381A (Msi2 AA). Following re-injection, the oocytes were allowed to rest for 1 hour before being stimulated with progesterone to induce maturation. The extent of cell cycle rescue for each condition was assessed when 50% of Msi2 WT injected oocytes had completed GVBD. The combined data for three independent experiments are shown; error bars represent S.E.M. (**c**) Cell lysates from one of the experiments in panel (b) were analyzed for expression of the GST-tagged Msi2 WT or Msi2 AA proteins (arrowhead). When 50% of the Msi2 WT oocytes had reached GVBD (GVBD_50_), they were segregated into those that had not (−) or had (+) completed GVBD. The filter was cropped to retain the 50–80 kD range, prior to western blotting. (**d**) Stage VI immature oocytes were injected with scrambled control antisense oligonucleotides (Con AS); Msi antisense oligonucleotides without (Msi AS) or with re-injection of RNA encoding *Xenopus* Msi2 or mutant Msi2 AA and subsequently stimulated with progesterone. Total RNA was prepared from immature stage VI oocytes (I), or Con AS when 50% of the oocytes had reached GVBD (GVBD_50_), segregated into those that had not (−) or had (+) completed GVBD. Similarly, RNA was prepared from Msi AS at the time when Con AS reached GVBD_50_, or Msi AS oocytes expressing Msi2 when they reached GVBD_50_, segregated into those that had not (−) or had (+) completed GVBD. Total RNA was also prepared from Msi AS oocytes expressing Msi2 AA, which did not mature, at the time when the Msi AS + Msi2 reached GVBD_50_. Progesterone-dependent polyadenylation (indicated by a retarded mobility shift) of the endogenous Mos, cyclin B5 and Msi1 mRNAs was assessed by RNA ligation coupled PCR.

The canonical form of the vertebrate Msi2 contains two serine/proline (S/P) motifs that are homologous with the sites critical for control of Msi1 function (Fig. 1a and refs 30 and 47). To directly assess the role of the conserved S/P motifs in regulating Msi2 function, we expressed a mutated form of the *Xenopus* Msi2 protein where serine 356 and 381 were changed to non-phosphorylatable alanine (Msi2 S356A/S381A) in oocytes in which the endogenous Msi1 and Msi2 had been functionally attenuated through use of Msi1/Msi2 antisense oligonucleotide injection. We tested the capacity of the mutant Msi2 S356A/S381A protein to rescue progesterone-stimulated oocyte maturation (Fig. 1b). In the absence of ectopic Msi2 expression, the Msi1/Msi2 antisense oligonucleotide injection abolished progesterone-stimulated progression to germinal vesicle (nuclear) breakdown (GVBD) and maturation (Fig. 1b, no rescue), as previously described^32^. While wild-type Msi2 effectively rescued oocyte maturation, expression of the mutant Msi2 S356A/S381A was unable to rescue this cell cycle defect indicating compromised function of the Msi2 S356A/S381A protein (Fig. 1b). Both mutant and wild-type proteins were expressed to equivalent levels (Fig. 1c). Oocyte maturation requires the polyadenylation-dependent translational activation of endogenous Msi target mRNAs including those encoding Mos, cyclin B5 and Msi1 itself^31–33^. While wild type Msi2 was able to rescue polyadenylation (indicated by retarded mobility of the PCR products), the Msi2 S356A/S381A mutant was unable to rescue activation of these mRNAs, consistent with a failure to rescue oocyte GVBD (Fig. 1d). These results illustrate two key facets of Msi2 function: conversion to an activator of translation requires S356/S381 site-specific regulatory phosphorylation; and Msi2 can regulate the same endogenous target mRNAs as the Msi1 isoform. This latter observation explains the partial redundancy of the Msi1 and Msi2 proteins in mediating *Xenopus* oocyte maturation^32^.

**Figure 2:**
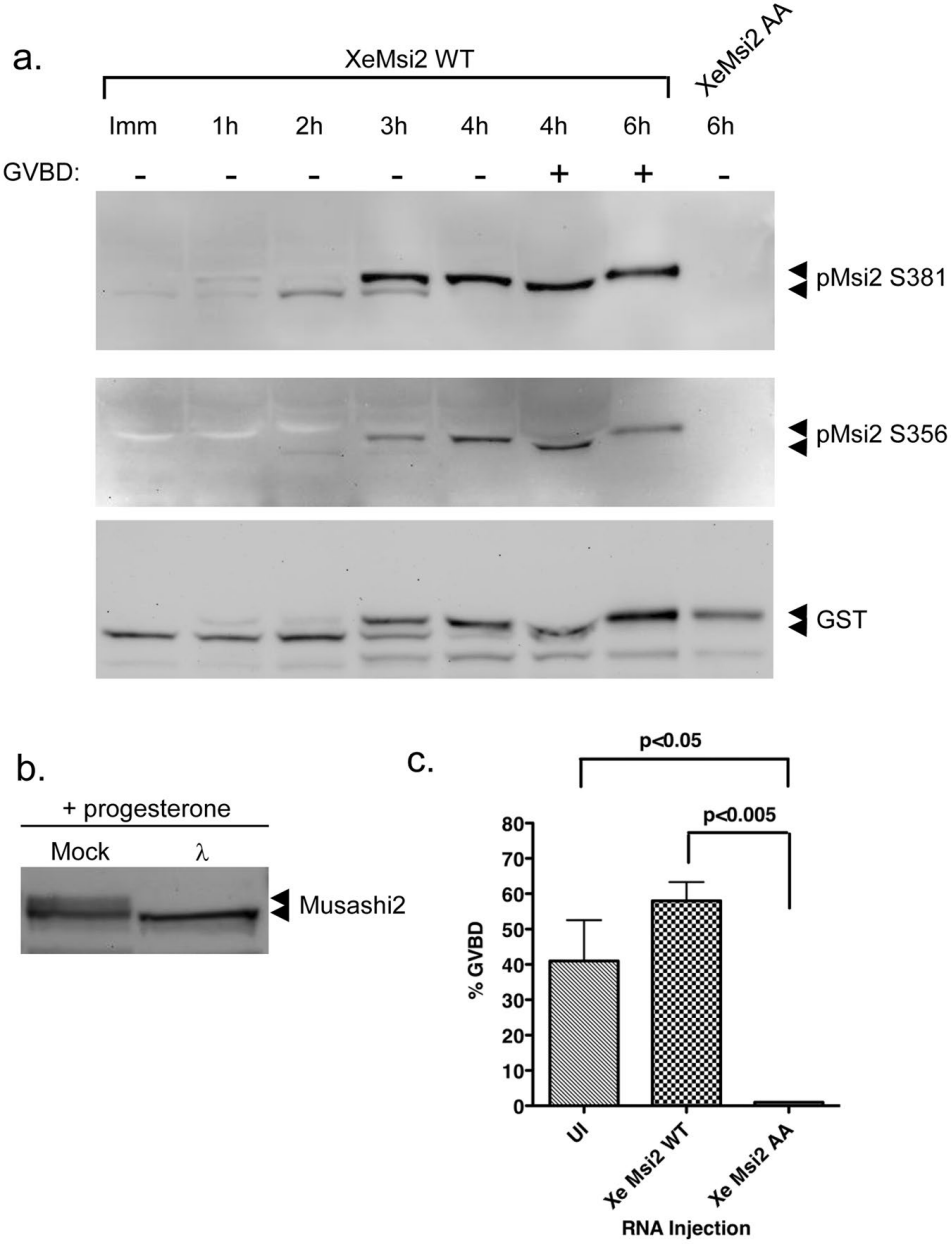
Progesterone-dependent phosphorylation of Msi2 during oocyte maturation. (**a**) Immature Stage VI oocytes were injected with RNA encoding GST-tagged *Xenopus* Msi2 (XeMsi2 WT) or mutant Msi2 S356A/S381A (XeMsi2 AA), incubated overnight to express the protein and subsequently stimulated to mature with progesterone. Protein lysates were prepared at the indicated times and the phosphorylation of S381 or S356 assessed with phospho-specific antisera as indicated. The lower panel represents a GST western blot to show the relative levels of the expressed proteins. XeMsi2 WT expressing oocytes reached GVBD_50_ after 4 hours and were segregated into those that had not (−) or had (+) completed GVBD. XeMsi2 AA oocyte samples were prepared 6 hours after progesterone treatment, but they had still not undergone GVBD. The filter was cropped to retain the 50–80 kD range, prior to western blotting. A representative experiment is shown. (**b**) Immature Stage VI oocytes were injected with RNA encoding GST-tagged *Xenopus* Msi2 (XeMsi2 WT) and incubated overnight. The oocytes were then stimulated to mature with progesterone. When the progesterone treated oocytes had completed GVBD, samples were prepared and either mock treated or incubated with λ phosphatase prior to analysis by GST western blotting. The filter was cropped to retain the 50–80 kD range, prior to western blotting. (**c**) 50–60 immature Stage VI oocytes were left untreated (UI) or injected with RNA encoding either GST-tagged XeMsi2 WT or XeMsi2 AA, incubated overnight and subsequently stimulated to mature with progesterone. The extent of GVBD in each cohort was assessed when 40–50% of the control (UI) oocytes reach GVBD. The results represent three independent experiments; error bars represent S.E.M.

Using Msi2 phospho-specific antisera (see Materials and Methods) and an over-expressed, GST-tagged form of *Xenopus* Msi2, we found phosphorylation of both S356 and S381 were significantly increased in a progesterone-dependent manner and occurred early, prior to GVBD (Fig. 2a). This early phase of maturation is specifically dependent upon Msi function^32, 33, 48^. The kinetics of phosphorylation were similar for both sites, with a low level of each phosphorylation event detectable at ~2 hour prior to GVBD with an abrupt increase in both sites ~1 hour prior to GVBD and subsequently maintained after GVBD (GVDB+) (Fig. 2a). The Msi2 protein underwent a mobility shift after progesterone stimulation prior to completion of GVBD. As expected, no site-specific phosphorylation was detect in the mutant Msi2 S356A/S381A protein (Fig. 2a, + progesterone, 6 hour time point), indicating the specificity of the antisera to their respective phosphorylated residues. The Msi2 protein exhibited a mobility retardation after progesterone stimulation prior to completion of GVBD and we note that the mutant Msi2 S356A/S381A protein exhibited a similar progesterone-dependent mobility shift. The mobility shift of the Msi2 protein, while independent of either S356 or S381 phosphorylation (Fig. 2a), was due to phosphorylation of other sites on the Msi2 protein since treatment of the lysate with lambda protein phosphatase abolished the observed mobility shift (Fig. 2b). Since these phosphorylation events were insufficient to mediate target mRNA translational activation or rescue oocyte GVBD (Fig. 1), the physiological significance of the mobility shift remains to be determined. Interestingly, in the absence of prior injection with Msi1/2 antisense oligonucleotides and unlike the wild-type Msi2, overexpression of the Msi2 S356A/S381A mutant functioned in a dominant negative manner to inhibit progesterone-stimulated maturation (Fig. 2c), suggesting the mutant may be sequestering signaling components or a co-factor required for oocyte maturation.

**Figure 3:**
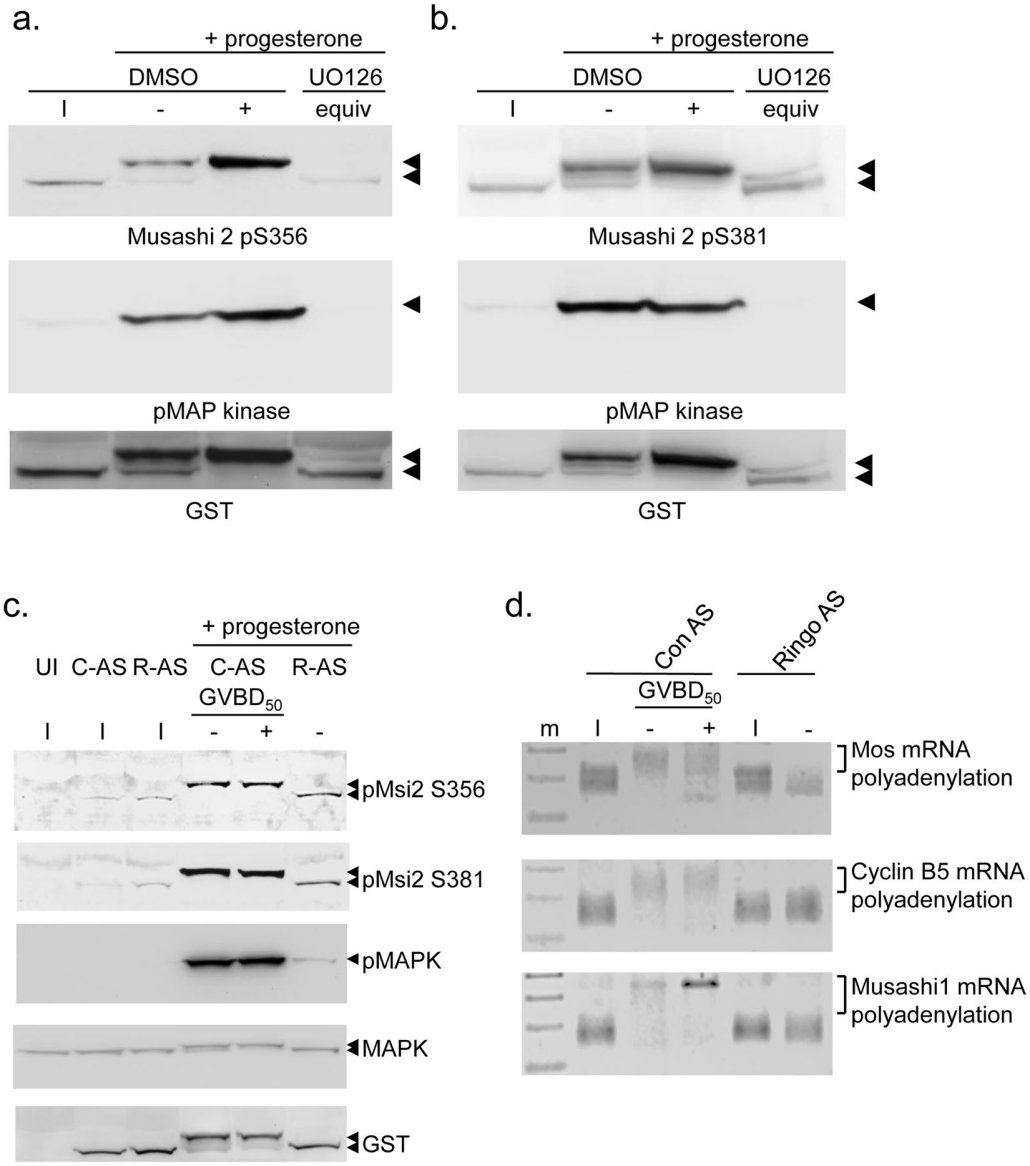
MAP kinase and Ringo signaling pathways mediate Msi2 S356 and S381 phosphorylation. (**a**) Immature oocytes (I) were injected with RNA encoding GST-tagged *Xenopus* Msi2 and incubated overnight to express the protein. The next morning the oocytes were split into two pools and either treated with DMSO (vehicle control) or U0126 for 1 hour prior to progesterone stimulation. When DMSO-treated oocytes reached GVBD_50_, oocytes were segregated into those that had not (−) or had (+) completed GVBD. UO126–treated oocyte lysates did not mature in response to progesterone and so time-matched (GVBD−) samples were prepared when DMSO treated oocytes reached GVBD50 (UO126 equiv). Lysates were probed with Msi2 S356 phospho-specific antisera, phospho-MAPK or GST antisera as indicated. The filter was cropped to retain the 50–80 kD range, prior to western blotting for the GST and pMsi2 S365 antibodies, and cropped to retain the 30–50 kD range for the phospho-MAPK western. (**b**) as (**a**), except probed with Msi2 S381 phospho-specific antisera. (**c**) Immature oocytes were co-injected with RNA encoding GST-tagged *Xenopus* Msi2 and either scrambled control or Ringo antisense oligonucleotides (C-AS or R-AS) and incubated overnight. Oocytes were then split into two pools and either left unstimulated (I) or stimulated with progesterone. Time matched protein lysates were prepared when progesterone stimulated C-AS oocytes reached GVBD_50_. C-AS oocytes were segregated into those that had not (−) or had (+) completed GVBD. Protein lysates were probed by western blotting with the indicated antibodies. The phospho-Msi2 images were over-exposed to demonstrate a low level of basal phosphorylation in immature oocytes. A lower exposure of these panels is shown in the Supplementary data. The filter was cropped prior to western blotting, as described for panel (a). In addition, the filter was cropped to retain the 30–50 kD range for the total MAPK western blot. (**d**) Progesterone-dependent polyadenylation of the endogenous Mos, cyclin B5 and Musashi1 mRNAs was assessed by RNA ligation coupled PCR in scrambled, control or Ringo antisense injected oocytes, essentially as described for Fig. 1d.

We next sought to determine which signaling pathway(s) contributed to the regulatory phosphorylation of Msi2. Our prior analysis of Msi1 regulatory phosphorylation implicated the sequential action of the Ringo/CDK signaling and subsequent p42 mitogen activated protein (MAP) kinase pathways^47^. Here, we found that treatment of oocytes with the MAP kinase signaling inhibitor, UO126, abolished MAP kinase activation and dramatically reduced progesterone-stimulated phosphorylation of both S356 and S381 sites on Msi2 (Fig. 3a and b). However, since low level phosphorylation of the S356 and S381 sites remained in UO126-treated oocytes, we also investigated the possible contribution of upstream, MAP kinase-independent pathways. Translation of the Ringo mRNA and activation of Ringo/CDK signaling is an early event in progesterone-stimulated oocyte maturation and precedes translational activation of the Mos mRNA^49^, which encodes the primary MAP kinase activator in oocytes^50–52^. Consistent with a contribution from this pathway, inhibition of Ringo/CDK signaling, through microinjection of Ringo antisense oligonucleotides^53^, was found to attenuate the levels of Msi2 S356 and S381 phosphorylation (Fig. 3c, R-AS). However, a low level of Ringo/CDK-independent, MAP kinase activity was still retained in Ringo antisense-treated oocytes (Fig. 3c). These results could suggest incomplete attenuation of Ringo/CDK signaling, leading to subsequent low level MAP kinase activation. However, injection of Ringo antisense oligonucleotides attenuated oocyte maturation and abolished progesterone-stimulated, Musashi-dependent polyadenylation and translational activation of endogenous Mos, cyclin B5 and Musashi1 mRNAs (Fig. 3d). We conclude that like Msi1, both MAP kinase and Ringo/CDK contribute to Msi2 regulatory phosphorylation. However, unlike the situation with Msi1, the precise order and relative contribution of MAP kinase and Ringo/CDK action on Msi2 S356 and S381 phosphorylation after progesterone stimulation remains to be established.

### Mammalian Msi2 function is also controlled by site-specific phosphorylation

**Figure 4:**
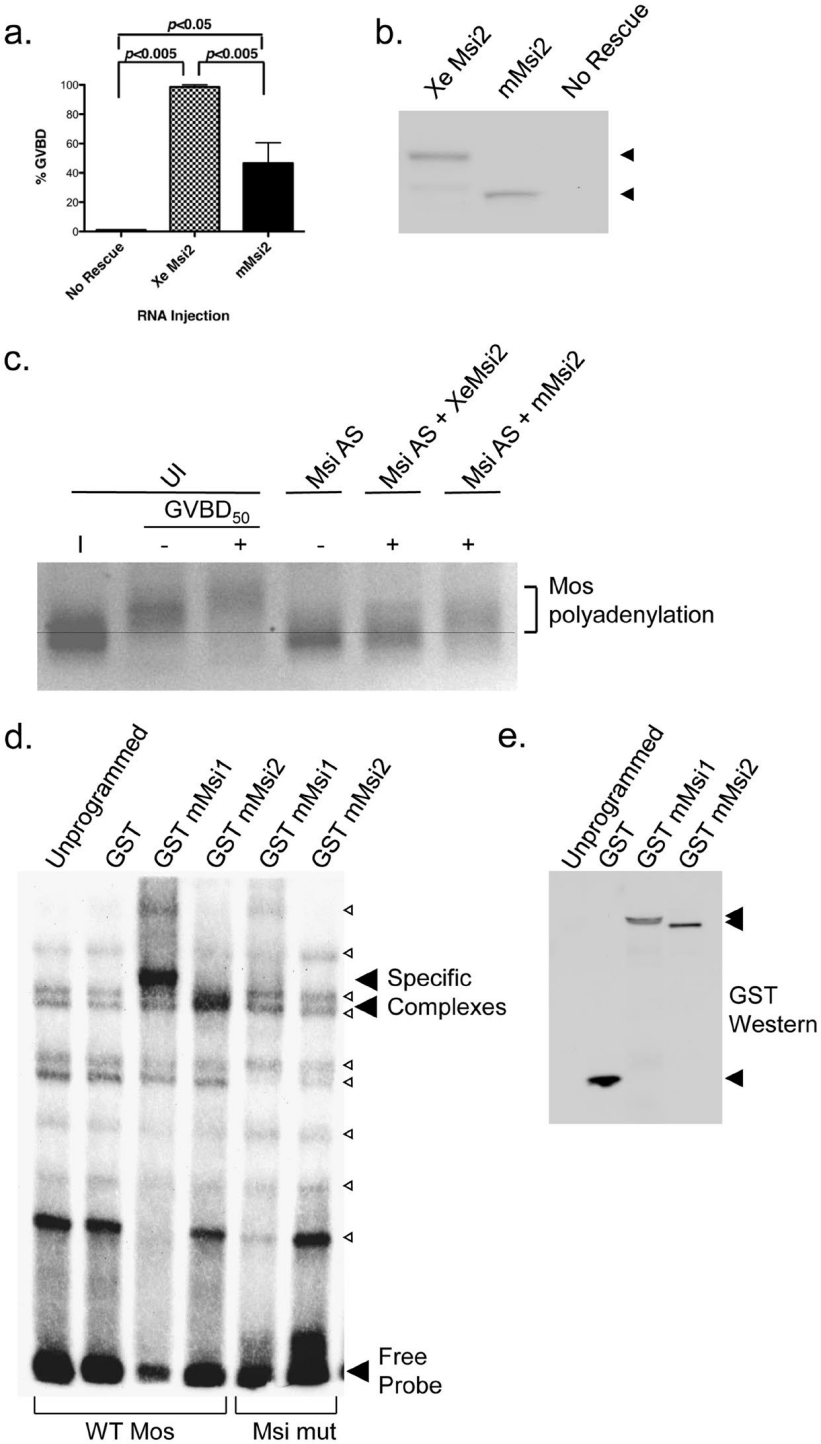
Mammalian Msi2 can direct mRNA translational activation and *Xenopus* oocyte maturation. (**a**) Msi1/2 antisense rescue assay, essentially as described in the legend to Fig. 1b, comparing cell cycle progression in GST-tagged *Xenopus* Msi2 and GST-tagged murine Msi2 expressing oocytes. Oocyte GVBD was scored when 100% of *Xenopus* Msi2 injected oocytes completed GVBD. The data represent the results of three independent experiments; error bars indicate S.E.M. (**b**) Equivalent cell lysates from one of the experiments in panel (a) were analyzed for expression of the GST-tagged *Xenopus* or murine Msi2 proteins (arrowheads). The filter was cropped to retain the 50–80 kD range, prior to western blotting. (**c**) Progesterone-dependent polyadenylation (retarded mobility shift) of the endogenous Mos mRNA was assessed by RNA ligation coupled PCR in Musashi antisense (Msi AS) injected oocytes, essentially as described for Fig. 1d, with either *Xenopus* Msi2 (XeMsi2) or murine Msi2 (mMsi2) rescue as indicated. (**d**) RNA EMSA using either a biotinylated Mos probe (WT Mos) with an intact Msi binding element (MBE) or a mutant Mos probe with a disrupted MBE (Msi mut), incubated with unprogrammed rabbit reticulocyte lysate or lysate expressing GST, GST murine Msi1 (mMsi1) or GST murine Msi2 (mMsi2), as indicated. Specific Msi1 and Msi2 complexes (solid arrowheads) form with the WT Mos probe but not the Msi mut probe. A number of additional non-specific bands are observed with unprogrammed lysate alone (open arrowheads). (**e**) Western blot of unprogrammed, GST, GST mMsi1 or GST mMsi2 expressing reticulocyte lysates used in the gel shift assay (**d**).

Expression of the murine Msi2 (mMsi2) protein in Msi1/2 antisense-injected oocytes also rescued progesterone-stimulated oocyte maturation (Fig. 4a), albeit with delayed kinetics relative to the *Xenopus* Msi2 ortholog. Since equivalent levels of the *Xenopus* and mMsi2 proteins were used (Fig. 4b), the differences in kinetics suggest that the mMsi2 may have compromised function in the heterologous oocyte assay. Nonetheless, when the behavior of the endogenous Mos mRNA was examined, both *Xenopus* Msi2 and mMsi2 proteins were able to direct polyadenylation and translational activation (Fig. 4c). mMsi2 was also able to direct polyadenylation and translational activation of the Cyclin B5 and Msi1 mRNAs (data not shown).

The ability of mammalian Msi2 to rescue endogenous mRNA polyadenylation, translational activation and oocyte maturation was due to an ability to interact directly with Msi1 target mRNAs. We utilized a biotinylated Mos wild-type UTR containing an intact Msi binding element (MBE), or a Mos UTR with a mutationally disrupted MBE ((through mutation of the MBE from AUAGU to AUccU, ref. 33) in RNA electrophoretic mobility shift assays (EMSA). GST-tagged forms of the murine Msi1 and Msi2 proteins both interacted specifically with the wild-type Mos UTR probe (Fig. 4d). No mMsi1 or mMsi2 binding was detected when the MBE-disrupted Mos UTR probe (Msi mut) was used, demonstrating that the interactions required an intact MBE. Moreover, the GST protein moiety failed to interact with the wild type Mos UTR probe, showing that the mobility shift specifically required mMsi1 or mMsi2 interaction with the RNA probe (Fig. 4d). Equivalent levels of GST, GST-tagged mMsi1 and GST-mMsi2 were used in the EMSA experiments (Fig. 4e).

**Figure 5:**
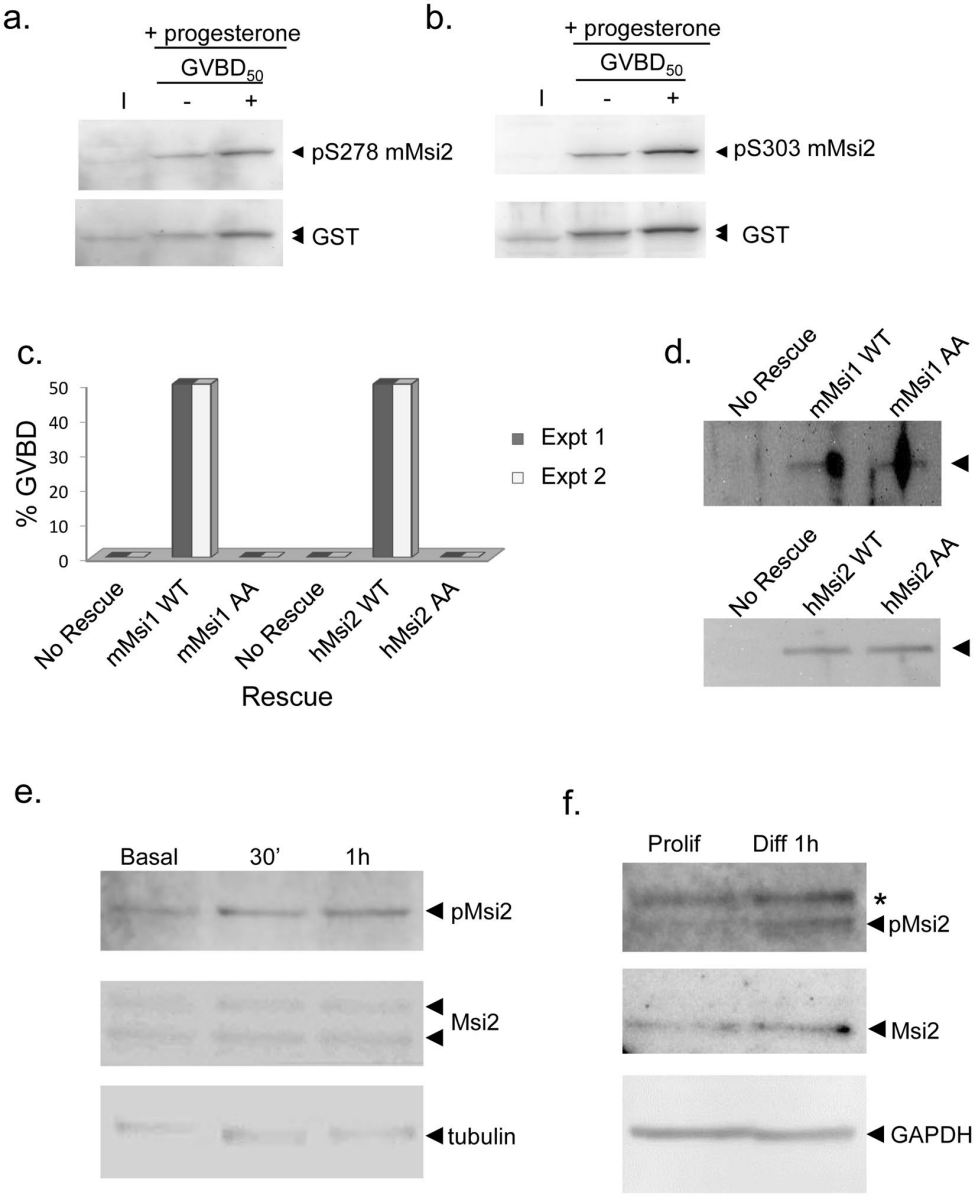
Regulatory phosphorylation controls mammalian Msi2 activity. (**a**,**b**) Immature oocytes were injected with RNA encoding GST-tagged murine Msi2 and incubated overnight to express the protein. The oocytes were then split into two pools and either left untreated or stimulated with progesterone. When stimulated oocytes reached GVBD_50_, oocytes were segregated into those that had not (−) or had (+) completed GVBD. Equivalent cell lysates from each condition were probed with phospho-specific antisera that recognizes S278 of the mammalian Msi2 protein (equivalent to S356 of *Xenopus* Msi2), or S303 (the equivalent of *Xenopus* Msi2 S381) or GST antisera to detect the expressed protein, as indicated. Mobility shifted forms of Msi2 are indicated by an upper arrowhead on the lower panels. The filter was cropped to retain the 50–80 kD range, prior to western blotting. (**c**) A Msi1/2 antisense rescue assay, essentially as described in Fig. 1b, comparing progression to GVBD in GST-tagged murine Msi1 (mMsi1 WT), S312A/S337A phosphorylation mutant mMsi1 (mMsi1 AA), human Msi2 (hMsi2 WT) or S278A/S303A phosphorylation mutant Msi2 (hMsi2 AA). Oocyte GVBD was scored when 50% of mMsi1 reached GVBD (for mMsi1 AA) or when 50% of hMsi2 reached GVBD (for hMsi2 AA). Two independent experiments are shown. (**d**) Lysate from unstimulated oocytes in panel (c) experiment 2 were analyzed by GST western blotting for expression of mMsi1 (upper panel) or hMsi2 (lower panel). The filter was cropped to retain the 50–80 kD range, prior to western blotting. (**e**) Western blot analysis of murine 32D cells prepared at different times during differentiation (30 mins and 1 hour, respectively) *vs*. basal proliferation conditions (Basal) probed with mammalian Msi2 S303 phospho-specific antisera (upper panel), Msi2 antisera, or tubulin. In 32D cells, the larger Msi2 isoform showed increased regulatory phosphorylation. The filter was cropped to retain the 30–50 kD range, prior to western blotting for Msi2 or cropped to retain the 40–60 kD range, prior to western blotting for tubulin. (**f**) Western blot analysis of human SHSY5Y cells undergoing proliferation (Prolif) or 1 hour after induction of differentiation (Diff) with retinoic acid. The Msi2 isoform showed increased regulatory S303 phosphorylation. A cross-reacting, non-specific band runs above the phosphorylated Msi2 isoform in the upper panel (asterisk). GAPDH serves as a loading control. The filter was cropped to retain the 30–50 kD range, prior to western blotting. The phospho-Msi2 image was over-exposed to more clearly demonstrate increased phosphorylation. A lower exposure of this panel is shown in the Supplementary data.

Western blot analyses demonstrated that the mMsi2 protein also underwent progesterone-stimulated phosphorylation of the homologous sites of regulatory phosphorylation (S278 and S303 in the mMsi2, the equivalent of *Xenopus* S356 and S381; Fig. 5a and b, respectively), suggesting a conserved role for phosphorylation-mediated regulation of Msi2 function. To directly verify this hypothesis, we tested the wild type human Msi2 (hMsi2), or a phosphorylation mutant human Msi 2 (hMsi2 S278A/S303A) in the Msi1/2 antisense rescue assay. While the wild type hMsi2 was able to rescue oocyte maturation in treated oocytes, similar to wild type murine Msi1 (mMsi1), neither the hMsi2 S278A/S303A nor mMsi1 S312A/S337A phosphorylation mutants were able to do so (Fig. 5c) even though equivalent levels of mutant and wild-type proteins were expressed (Fig. 5d). We conclude that site-specific regulatory phosphorylation of the canonical Msi2 protein isoform is an evolutionarily conserved mechanism to promote de-repression and translation of target mRNAs.

To determine if Msi2 undergoes regulatory phosphorylation in mammalian cells, we assessed Msi2 phosphorylation status during the transition from proliferation to differentiation using an immortalized murine myeloblast-like cell line, 32D as well as a human neuroblastoma cell line (SHSY5Y). The 32D cell line undergoes differentiation after switching proliferating cells from a culture containing interleukin-3 (IL-3) to one containing granulocyte-colony stimulating factor (G-CSF)^54^. We observed enhanced Msi2 regulatory S303 phosphorylation during 32D cell differentiation (Fig. 5e). This increased phosphorylation was a rapid response, detectable within 30 minutes of differentiation induction. We observed a low level basal S303 phosphorylation of Msi2 in proliferating human SHSY5Y neuroblastoma cells grown in non-adherent spheroid culture and an increase in Msi2 regulatory phosphorylation in response to retinoic acid induced differentiation of SHSY5Y cells (Fig. 5f). This increased Msi2 phosphorylation is consistent with the observed de-repression of Msi target mRNAs as SHSY5Y cells undergo differentiation^30^.

### Alternative splicing generates a human Msi2 isoform that is refractory to regulatory phosphorylation

**Figure 6:**
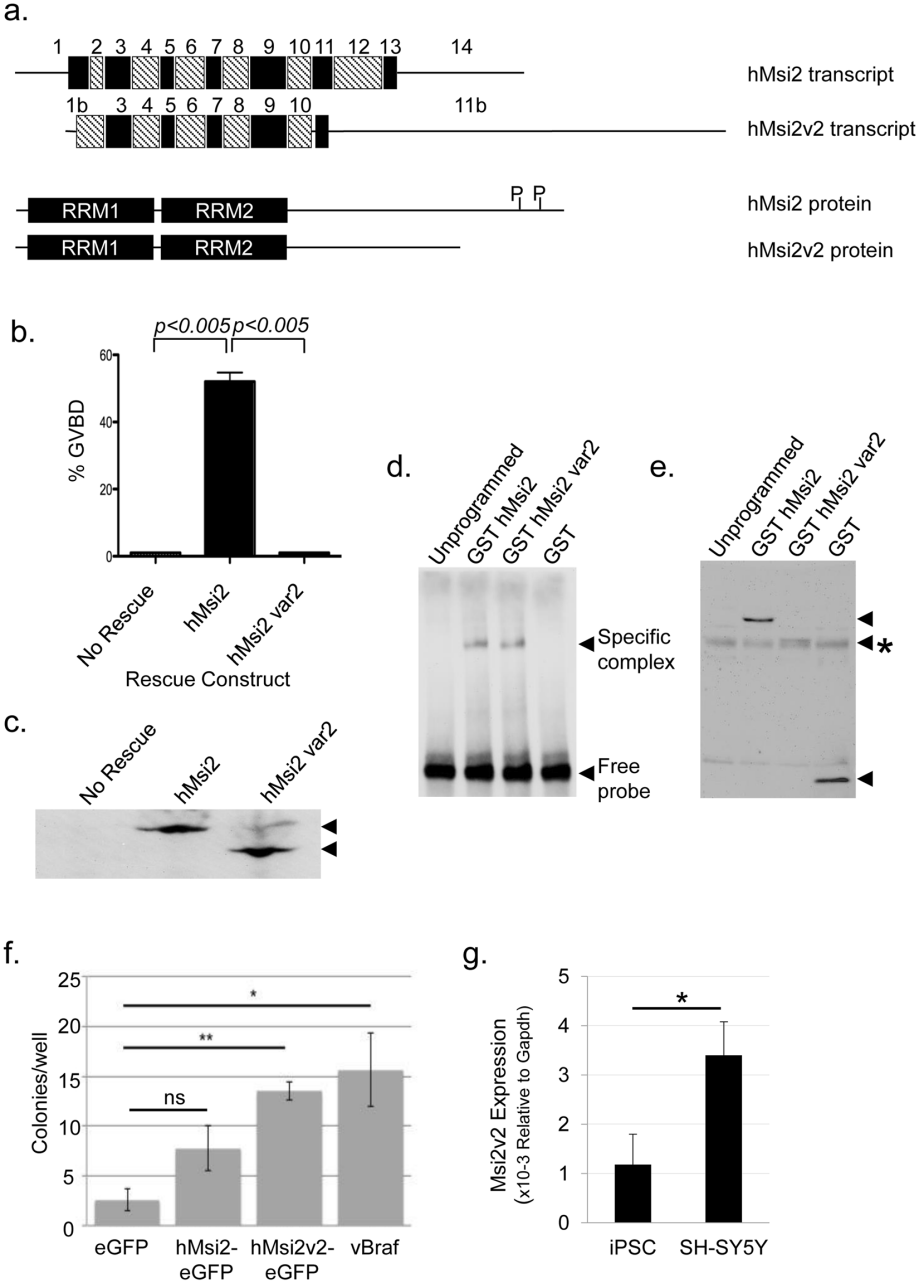
The Msi2 variant 2 promotes transformation of NIH3T3 cells. (**a**) Schematic showing alternative exon usage between the canonical human Msi2 isoform (hMsi2) and the variant 2 isoform (hMsi2v2). Substitution of exons 1 and 2 for exon 1b in hMsi2v2 results in a novel 17 amino acid N-terminal domain. Exons 3–11 are shared between the two isoforms, but use of the alternate 11b exon results in a unique terminal 13 amino acid sequence in the truncated C-terminal domain. Lower panel show schematic alignment of the canonical human Msi2 protein (Accession NP_620412) with the alternatively spliced human Msi2 variant 2 protein (Accession NP_733839). RRM1 and RRM2, RNA recognition motifs 1 and 2; P, indicates position of the sites of regulatory phosphorylation in the canonical Msi2 isoform. (**b**) A Musashi antisense rescue assay comparing progression to oocyte GVBD in GST-tagged hMsi2 or hMsi2 variant 2 (Msi2 var2). Rescue was scored when 50% of hMsi2-injected oocytes reached GVBD. Data shown are from three independent experiments; error bars represent S.E.M. (**c**) Lysates from unstimulated oocytes in panel (b) were analyzed by GST western blotting. The filter was cropped to retain the 50–80 kD range, prior to western blotting. (**d**) RNA EMSA using a biotinylated murine Pou1f1 probe with consensus MBE (GUAGG), incubated with unprogrammed rabbit reticulocyte lysate or lysate expressing GST fusion proteins: GST human Msi2 (hMsi2), GST human Msi2 variant 2 (hMsi2 var2) or the GST moiety alone, as indicated. A specific Msi2 complex (solid arrowhead) is indicated. (**e**) A GST western blot of the reticulocyte lysates used in the gel shift assay (**d**). GST proteins are indicated by arrowheads. The hMsi2 var2 runs just above a non-specific band (asterisk). (**f**) 3000 NIH3T3 cells were plated per well of a 24 well ultra-low adherence tissue culture dish and transfected in quadruplicate with the indicated expression construct. Colony growth was scored at day 10 after transfection. The data are combined from three independent transfection experiments. *p < 0.05; **p < 0.01, ns, not significant; n=3. (**g**) qRT-PCR showing expression levels of Msi2 var2 RNA in human iPSCs and SH-SY5Y cells relative to level of GAPDH RNA. The combined data from three independent experiments are shown. *p < 0.05.

In addition to the canonical Msi2 (RefSeq Accession NM_138962), humans are predicted (Ensembl, ref. 55) to express a novel alternatively spliced variant of Msi2, termed variant 2 (RefSeq Accession NM_170721). The Msi2 variant 2 isoform encodes a truncated protein lacking the two sites of regulatory phosphorylation present in exon 12 of the longer, canonical Musashi2 protein due to alternative splicing of an alternative exon 11 (Fig. 6a, designated 11b). Exon 11b originates within the intronic sequence between exons 10 and 11 of the canonical Msi2 (NM_138962). The Msi2 variant 2 isoform also has an N-terminal 17 amino acid substitution (MADLTSVLTSVMFSPSS) replacing the first 21 amino acids of the canonical Msi2 protein (MEANGSQGTSGSANDSQHDPG). This substitution is due to alternative splicing that replaces the first and second exon found in the canonical isoform with an alternative first exon (designated 1b) that originates within intron 1–2 of the Msi2 (NM_138962) transcript. In all other respects, the two Msi2 proteins have identical RNA recognition motifs (RRMs), and identical C-terminal domain up through exon 10. The Msi2 variant 2 isoform possesses a novel 13 amino contiguous C-terminal sequence (DYLPVSQDIIFIN) not found in the canonical human Msi2 isoform. Using this sequence in a BLAST search (NCBI, tblastn), predicted orthologs of the human Msi2 variant 2 were identified in a number of vertebrate species. Curiously, no murine ortholog of Msi2 variant 2 was identified, although several alternatively spliced truncated murine Msi2 isoforms are predicted that lack the C-terminal sites of regulatory phosphorylation (Ensembl, ref. 55). Analysis of human RNA-seq data available through the Illumina human Bodymap 2.0 project, indicate that Msi1 was predominantly expressed in brain, ovary and testes but was not detected in skeletal muscle or blood (Table 1). Msi2 was more highly expressed than Msi1 in all tissues examined, and was expressed in skeletal muscle and blood. The Msi2 variant 2 isoform was predominantly expressed in brain and thyroid, out of the 16 tissues represented in the dataset. Proportional to the canonical Msi2 transcript, the levels of the alternatively spliced Msi2 variant 2 were most elevated in brain and liver.

**Table 1:** Normalized expression of Msi1, Msi2 and Msi2 variant 2 transcripts in sixteen human tissues.

Sample name	Tissue type	FPKM of Msi1 (NM_002442)	FPKM of Msi2v2 (NM_170721)	FPKM of Msi2 (NM_138962)	Ratio FPKM Msi2v2/Msi2
ERR030888	Adipose	0.127	0.304	4.119	0.074
ERR030889	Adrenal	0.292	0.616	4.330	0.142
ERR030890	Brain	3.604	6.406	17.807	0.360
ERR030891	Breast	0.606	0.367	4.877	0.075
ERR030892	Colon	1.313	0.414	4.546	0.091
ERR030893	Kidney	0.307	1.925	14.115	0.136
ERR030894	Heart	1.031	1.358	35.321	0.038
ERR030895	Liver	0.025	1.071	4.276	0.250
ERR030896	Lung	0.668	0.753	7.446	0.101
ERR030897	Lymph node	0.061	1.543	8.536	0.181
ERR030898	Prostate	1.037	0.983	6.186	0.159
ERR030899	Skeletal muscle	0.000	0.661	30.939	0.021
ERR030900	Leukocyte (blood)	0.000	0.850	7.334	0.116
ERR030901	Ovary	3.481	0.620	12.524	0.050
ERR030902	Testis	5.577	1.232	6.753	0.182
ERR030903	Thyroid	0.265	4.018	19.224	0.209

Focused gene expression of Msi1 (NM_002442), Msi2 variant 2 (NM_170721) and Msi2 (NM_138962) were assessed using publically available transcription profiling (RNA-Seq) of 16 individual human tissues from the Illumina Human Body Map 2.0 dataset). Expression is shown as fragments per kilobase of exon per million fragments mapped (FPKM). Msi1 was predominantly expressed in brain, ovary and testes but was not detected in skeletal muscle or blood. Msi2 was more highly expressed than Msi1 in all tissues examined, as was expressed in skeletal muscle and blood. The proportion of Msi2 variant 2 to Msi2 was highest in brain and liver (36 and 25%, respectively).

Given the requirement for regulatory phosphorylation to promote translational activation of Msi2 target mRNAs, we predicted that the human Msi2 variant 2 isoform would be unable to rescue oocyte maturation when endogenous Msi function was ablated. Indeed, the expressed Msi2 variant 2 failed to mediate progesterone-stimulated maturation in the *Xenopus* oocyte Msi1/Msi2 antisense rescue assay (Fig. 6b) even though equivalent levels of the canonical hMsi2 and the hMsi2 variant 2 proteins were expressed (Fig. 6c). Using RNA EMSA, we confirmed that the hMsi2 variant 2 was able to bind to a target mRNA with similar efficacy as the canonical hMsi2 protein (Fig. 6d and e). These findings indicate that the inability of the alternatively spliced Msi2 variant 2 isoform to mediate oocyte maturation is most likely due to the fact the Msi2 variant 2 protein evades the C-terminal regulatory phosphorylation necessary for de-repression and/or activation of target mRNA translation.

We speculated that the inability of the Msi2 variant 2 isoform to undergo de-repression and translation of target mRNAs in response to regulatory signaling may confer pro-oncogenic properties to the protein. To test this hypothesis, NIH3T3 cells (which lack endogenous Musashi expression^25^) were transfected with eGFP-tagged forms of the canonical human Msi2 or the Msi2 variant 2 or with the eGFP moiety alone and grown on non-adherent plates in the presence of selection for the expression plasmid. One day after transfection, cells were examined for eGFP fluorescence and we verified equivalent transfection efficiency of all Msi2 expression plasmids. After 10 days in culture, anchorage-independent colony growth was scored (Fig. 6f). In these assays, an oncogenic form of BRaf, encoding the C-terminal kinase domain (vBRaf, ref. 56) was used as a positive control. Expression of Msi2 variant 2 and vBRaf conferred significant enhancement of anchorage-independent colony growth when compared to the eGFP moiety or the canonical Msi2 isoform. To determine if endogenous expression of Msi2 variant 2 is associated with loss of growth control, we examined levels in human induced pluripotent stem cells (iPSCs) and in the transformed human SH-SY5Y (neuroblastoma) cell line. Consistent with a pro-transformation function, qRT-PCR analyses revealed that the levels of the Msi2 variant2 were up-regulated in SH-SY5Y cells compared to iPSCs (Fig. 6g).

To more directly assess the possible relationship between Msi2 variant 2 expression and cancer, we assessed the relative expression levels of both canonical Msi2 and Msi2 variant 2 transcript expression in 12 different cancers, using publically available RNA-seq data from the Cancer Genome Atlas (TCGA) via the Cancer RNA-seq Nexus web portal^57^. Of 12 cancer types analyzed, Msi2 variant 2 transcript expression was upregulated in 9 cancer types across some, if not all stages of disease progression (Table 2 and Supplementary Table S1). Of these 9 cancer types, the canonical Msi2 transcript was also upregulated in 7, by a similar magnitude (e.g. breast invasive carcinoma Table 2). In 2 cancer types, head and neck squamous cell carcinoma (Supplementary Table S1) and uterine corpus endometrial carcinoma (Table 2), a 2–3 fold increase in Msi2 variant 2 transcript levels occurred without a significant increase in the canonical Msi2 transcript. No increased expression of Msi2 or Msi2 variant 2 was observed in prostate adenocarcinoma, thyroid carcinoma or kidney renal clear cell carcinoma (Supplementary Table S1) when compared to normal adjacent tissue.

**Table 2:** Differential expression of Msi2 and Msi2 variant 2 transcripts in human cancer vs normal adjacent tissue.

Cancer type	>10 samples?	Msi2		Adjusted *p* value	Msi2v2		Adjusted *p* value
		Cancer (TPM)	adjacent normal (TPM)		Cancer (TPM)	adjacent normal (TPM)	
**Breast Invasive Carcinoma, 1191 samples, 13 subsets**							
Stage I (90) vs Adjacent Normal (111)	yes	20.32	9.64	1.13E-08	3.84	1.12	2.27E-16
Stage IA (84) vs Adjacent Normal (111)	yes	17.63	9.64	1.80E-05	3.8	1.12	8.97E-15
Stage IB (9) vs Adjacent Normal (111)	no						
Stage II (3) vs Adjacent Normal (111)	no						
Stage IIA (360) vs Adjacent Normal (111)	yes	18.69	9.64	1.81E-09	3.64	1.12	1.07E-50
Stage IIB (249) vs Adjacent Normal (111)	yes	19.88	9.64	1.10E-09	3.67	1.12	3.85E-41
Stage IIIA (152) vs Adjacent Normal (111)	yes	19.69	9.64	6.46E-10	3.62	1.12	6.54E-34
Stage IIIB (29) vs Adjacent Normal (111)	yes	26	9.64	4.16E-04	3.88	1.12	1.22E-08
Stage IIIC (65) vs Adjacent Normal (111)	yes	17.43	9.64	1.13E-04	2.91	1.12	6.78E-11
Stage IV (22) vs Adjacent Normal (111)	yes	12.91	9.64	ns	2.76	1.12	4.08E-04
Stage X (14) vs Adjacent Normal (111)	yes	25.72	9.64	1.98E-03	3.85	1.12	1.94E-04
Metastatic Stage IIB (3) vs Adjacent Normal (111)	no						
**Uterine Corpus Endometrial Carcinoma, 580 samples**							
Tumor (545) vs adjacent normal (35)	yes	7.73	5.9	ns	2.56	1.11	4.46E-08

The CRN web portal (http://syslab4.nchu.edu.tw/) was used to interrogate TCGA RNA-seq datasets corresponding to 12 subtypes of invasive breast carcinoma and adjacent normal tissue, as well as tumor or normal adjacent TCGA RNA-seq datasets for endometrial carcinoma. The number of total RNA-seq samples are indicated for each condition in parentheses. For each comparison, the RNA-seq expression data were searched for Msi2 expression and the relative expression of the canonical Msi2 (uc002uiz) or Msi2 variant 2 (uc002iva) transcripts determined if the cancerous and adjacent normal data were derived from at least 10 samples as indicated (yes or no). Differential expression of Msi2 or Msi2 variant 2 (Msi2v2) in cancerous tissue vs adjacent normal tissue was not considered significant (ns) if the adjusted *p* value was >0.01. TPM, transcripts per million. Analyses of 10 additional cancers are summarized in Supplemental Table S1.

## Discussion

We demonstrate that canonical Msi2 function is controlled through site-specific regulatory phosphorylation. Regulatory control of *Xenopus* Msi2 function is mediated through both the Ringo/CDK and the MAP kinase signaling pathways. Although the regulatory phosphorylation sites reside within S/P motifs, it remains to be determined if Ringo/CDK and/or MAP kinase directly phosphorylate Msi2, or if intermediary kinases are involved. Physiologically, Msi2 phosphorylation is required for target mRNA de-repression and translational activation as a mutant form of Msi2 that cannot undergo regulatory phosphorylation is unable to mediate oocyte maturation. We observed a similar regulatory phosphorylation of mammalian Msi2 when ectopically expressed in *Xenopus* oocytes, as well as the endogenous mammalian Msi2 in murine 32D myoblastic cells and human SH-SY5Y neuroblastoma cells in response to extracellular signals that induce cell differentiation. These findings suggest that regulatory phosphorylation of Msi2 serves an evolutionarily conserved role in promoting cell cycle exit and cell differentiation. It will be interesting in the future to determine if the increase in Musashi2 regulatory phosphorylation is transient or if the phosphorylated Musashi2 species is sustained throughout the differentiation process.

The mechanism by which C-terminal regulatory S356 and S381 phosphorylation of Msi2 results in a functional switch to promote translation of target mRNAs is unknown. One potential mechanism could involve the modulation of target mRNA 3′ end polyadenylation, a key process for the translational activation of mRNAs. In support of this role, we have recently identified the Germline Development Defective-2 (GLD2) poly[A] polymerase as a *Xenopus* Msi2 interacting protein^45^. However, the interaction was observed to be independent of progesterone stimulation indicating that regulatory phosphorylation does not play a direct role in Msi2-GLD-2 association. The phosphorylation of Msi2 may serve an alternate role to facilitate activation of associated GLD2 function, or may effect a remodeling of other co-associated proteins independently of GLD2, to promote de-repression and translation of target mRNAs.

In view of the apparent requirement for Msi2 phosphorylation to promote target mRNA translation, we were intrigued by the expression of an alternatively spliced form of Msi2 that lacks the sites of regulatory phosphorylation and is compromised in ability to switch from a repressor to an activator of target mRNA translation. The alternatively spliced Msi2 variant 2 is expressed more highly in SH-SY5Y tumor cells than in normal iPSCs suggesting an association between expression of Msi2 variant 2 and cell transformation (Fig. 6g). In this regard, overexpression of the Msi2 variant 2, but not of Msi2 (variant 1) was able to confer significant anchorage-independent growth to cells (Fig. 6f). Moreover, interrogation of publicly available cancer RNA-seq data revealed significant upregulation of Msi2 variant 2 transcript expression in tumor vs normal control samples in 9/12 analyzed cancer types (Table 2, Table S1). Although these results suggest a correlation between increased Msi2 variant 2 expression and a subset of cancers, Msi2 variant 2 is expressed at quite low levels and so independent validation will be necessary to confirm these observations.

Alternative splicing of the mammalian Msi1 and Msi2 isoforms, involving inclusion or exclusion of internal exons has been previously observed^58–60^, although the reported isoforms all retain the two sites of identified C-terminal regulatory phosphorylation. The murine Msi1 and Msi2, as well as the human Msi1 and Msi2 genes encode the two sites of regulatory phosphorylation within a single exon. In murine embryonic stem cells (mESCs), two alternatively spliced murine Msi2 isoforms (both retaining the sites of regulatory phosphorylation) possess non-redundant activities, presumably due to differences in their ability to interact with specific cellular co-factors^60^. The alternatively spliced variant 2 form of human Msi2 differs from these examples since it has distinct N- and C-terminal amino acid sequences from the canonical Msi2 isoform. Of note, the substitution of the N-terminal sequence does not always correlate with alternative splicing of the C-terminal domain, when examined across species. The human Msi2 variant 2 has replaced exons 1 and 2 with an alternative exon 1b, and exon 12 (which encodes both sites of regulatory phosphorylation), exon 13 and exon 14 with an alternative exon 11b that results in a truncated protein (see Fig. 6a). A number of predicted truncated forms of murine Msi2 exist that retain both RRMs but lack the C-terminal phosphorylation sites, that would be predicted to behave in a manner analogous to the human Msi2 variant 2 and evade signals that switch canonical Msi1 and Msi2 function from repression to allow translation of target mRNAs. Interestingly, alternative splicing of the zebrafish Msi2b transcript generates several C-terminally truncated proteins that lack the two sites of regulatory phosphorylation and which may possess distinct functional properties^61^.

We propose that alternative splicing to generate C-terminally truncated Msi2 variant isoforms that retain sequence-specific RNA binding activity and potential to repress target mRNAs may enforce suppression of weak differentiation signals, an important consideration with regard to both stem/progenitor cell homeostasis and cell transformation. It is thus anticipated that repression of target mRNA translation would be exerted by Msi2 variant 2 even under conditions in cells where the canonical Msi1 and Msi2 isoforms are regulated through phosphorylation. Since de-repression of Msi target mRNAs is required for cell cycle exit and differentiation, alternate mechanisms must exist to block Musashi2 variant 2 function under conditions of physiological differentiation. The characterization of such alternative regulatory pathways is an important avenue for future investigation.

To date, no FDA approved pharmacological inhibitors of Msi function are available. An impediment to the therapeutic control of pathological Msi activity is a lack of understanding of the mechanisms by which Msi exerts translational control and the regulatory processes that impinge upon Msi function. We have now defined a common mechanism for regulation of canonical Msi1 and Msi2 isoform function, involving phosphorylation of two conserved sites in the C-terminal domain of the proteins. This phosphorylation is necessary to promote translation of target mRNAs during cell cycle exit in the *Xenopus* oocyte, and occurs during differentiation of mammalian cells. In addition, we have characterized a novel alternatively spliced Msi2 isoform that lacks these phosphorylation sites and is pro-oncogenic. Our findings reveal shared Msi1 and Msi2 regulatory properties that can be exploited therapeutically, but also highlight the unexpected complexity and compounding problem of Msi isoform-specific functional control.

## Methods

### Plasmid Construction

Plasmids encoding GST *Xenopus* Msi1 (xMsi1)^33^, GST-mMsi1^29^, GST-mMsi2^45^, SRα vBRaf^56^ have been previously described.

pXen XeMsi2. *Xenopus* Msi2^62^ was amplified from a cDNA plasmid provided by Dr. Martha Rebbert to include 5′ Cla1 and 3′ Xho1 restriction sites. The digested PCR fragment was cloned into pXen2^63^ to generate an in-frame GST tagged *Xenopus* Msi2.

pXen XeMsi2 S356A/S381A - Serines 356 and 381 of the XeMsi2 sequence were changed to alanine by site-directed mutagenesis (Quikchange II, Agilent Technologies), using pXen XeMsi2 as a template.

pXen hMsi2. Human Msi2 (Accession NM_138962, Open Biosystems) was PCR amplified to include a 5′ Cla1 site and a 3′ BamH1 site and cloned into Cla1/BamH1 digested pXen2.

pXen hMsi2 S278A/S303A, pXen mMsi1 S312A/S337A were generated by Quikchange site-directed mutagenesis using the wild-type plasmids as a template.

pXen hMsi2v2. Human Msi2 variant 2 (Accession NM_170721, Origene) was PCR amplified to include a 5′ Cla1 site, and a 3′ Xho1 site and cloned into Cla1/Xho1 digested pXen2.

hMsi2 eGFP and hMsi2v2 eGFP. hMsi2 and hMsi2v2 were PCR amplified to include a 5′ HindIII site and a 3′ BamH1 immediately prior to the STOP codon and cloned into HindIII/BamH1 digested pEGFP-N1 (Clontech) to generate an in-frame C-terminal eGFP fusion protein.

All cloned inserts and introduced mutations were verified by DNA sequencing.

### Oocyte culture and microinjections

Xenopus oocytes were isolated and cultured as described previously^64^. Oocytes were induced to mature with 2 µg/ml progesterone^65^. The rate of germinal vesicle breakdown (GVBD) was scored morphologically by observing the appearance of a white spot on the animal pole. Because oocytes from different frogs mature at different rates, the culture times were standardized between experiments to the time taken for 50% of oocytes to undergo GVBD. mRNA for oocyte injection was made by *in vitro* transcription using SP6 RNA Polymerase (Promega), as previously described^66^. Typically 23ng RNA was injected into each oocyte^45^. Where indicated, oocytes were pre-treated with 50 μM MEK inhibitor, UO126 (Promega) or DMSO vehicle for 30 minutes. At least 40 oocytes were scored for each condition, and all experiments were routinely repeated on three separate occasions. Where indicated, when 50% of control oocytes had reached GVBD, oocytes were segregated into those that had not (−) or had (+) completed GVBD. For all protein and RNA extractions, pools of 5–10 oocytes were used for each sample point. In all cases, oocytes were lysed in 10 μl NP40 lysis buffer/oocyte, and 0.5 oocyte equivalents loaded per lane^65^. Animal protocols were approved by the UAMS Institutional Animal Care and Use committee, in accordance with Federal regulations.

### RNA Electrophoretic mobility shift assay (EMSA)

GST fusion proteins were *in vitro* transcribed/translated using TNT SP6-coupled Reticulocyte Lysate System (Promega) from pXen1 (for GST), or pXen plasmids encoding the indicated Msi protein. 5′ biotin-labeled RNA oligonucleotide probes were synthesized by Integrated DNA Technologies and corresponded to the last 50 nucleotides of the *Xenopus* Mos 3′ UTR (Accession NM_001088094), a mutant Mos 3′ UTR encoding a disruption of the consensus MBE^33^ or the last 90 nucleotides of the murine Pou1f1 3′ UTR which encodes a consensus MBE (Accession BC061213). Binding reactions, electrophoresis and probe detection were performed as described^33^.

### Polyadenylation assays

cDNAs for polyadenylation assays were synthesized using RNA ligation-coupled PCR as described previously^67^. The increase in PCR product length (retarded mobility) is specifically due to extension of the poly[A] tail^67, 68^. The primers for endogenous Mos, cyclin B5 and Msi1 mRNAs have been described previously^31–33^. Verification of PCR product identity was performed by DNA sequence analysis.

### Antisense oligodeoxynucleotide injections

Antisense oligodeoxynucleotides targeting endogenous Msi1 and Msi2 mRNAs, Ringo mRNA or a randomized control oligonucleotide sequence 5′-TAGAGAAGATAATCGTCATCTTA-3′ were employed as previously described^32, 53^. Msi AS injected oocytes failed to undergo progesterone-stimulated maturation (GVBD) in the absence of expressed Msi protein. No inhibition of progesterone-stimulated maturation was observed with scrambled control antisense oligonucleotides.

### Cell culture

The murine IL-3-dependent 32D hematopoietic cell line, human SH-SY5Y, and human induced pluripotent stem cells (hiPSCs, KYOUDXR0109B) were originally obtained from ATCC (Manassas, VA). 32D cells were maintained in RPMI 1640 medium supplemented with 10% heat-inactivated fetal bovine serum (FBS), 100 U/ml penicillin and 100 μg/ml streptomycin (Atlanta Biologicals, Norcross, GA), and 5 ng/mL recombinant murine IL-3 (PeproTech, Rocky Hill, NJ). For inducing 32D cell differentiation, cells were washed with HBSS to remove IL-3, and 500,000 cells were plated on Day 0 in RPMI 1640 containing 10% FBS, 1% penicillin-streptomycin, and 40 ng/mL recombinant murine G-CSF (PeproTech, Rocky Hill, NJ). At the indicated time points, cells were harvested, cytospun, and then Wright’s-stained (EMD Chemicals) for analysis of nuclear morphology to determine the extent of granulocytic differentiation^54^. SHSY5Y human neuroblastoma cells were cultured in DMEM/F12 medium with 10% fetal bovine serum (Thermo Fisher Scientific/Gibco). Human iPSCs were cultured in Essential 8 Medium (Thermo Fisher Scientific/Gibco), as per the supplier's protocol.

### Quantitative real time PCR assays

Cell RNA was isolated using the Maxwell 16 LEV simplyRNA tissue kit (Promega; AS1280) and 50 ng RNA was utilized with RNA-to-Ct Power SYBR PCR master mix with a QuantStudio 12K Flex system (Thermo Fisher Scientific/Applied Biosystems), as per suppliers protocol. Three independent experiments were performed and each sample was analyzed in triplicate. Samples were normalized to *Gapdh* expression (Qiagen QuantiTect Primer Assay), and relative expression values were determined by the QuantStudio 12K Flex Software version 1.2.3 using the Δ-Δ-cycle threshold method^69^. Msi2 variant 2 sense primer: GAGGGTTTGGCTTTGTCACTTTTG; Msi2 variant 2 antisense: AATTATGTCTTGTGAAACCGGC. Sequencing of qPCR products confirmed the identity of the expressed Musashi2v2 isoform.

### Bioinformatic analyses

We used the publically available transcription profiling (RNA-Seq) of 16 individual human tissues (Illumina Human Body Map 2.0 dataset) to find the tissues where Msi1, Msi2 and Msi2 variant 2 transcripts are expressed (Accessions NM_002442, NM_138962 and NM_170721, respectively). FASTQ files were downloaded from the EMBL-EBI European Nucleotide Archive (http://www.ebi.ac.uk/ena/data/view/ERP000546). To estimate abundances at the gene-level and isoform-level we used the Tophat (v2.0.12)^70^ and Cufflinks (v2.2.1)^71^ software tools. Quality control for the reads was implemented using the fastx-toolkit (http://hannonlab.cshl.edu/fastx_toolkit). The quality of the reads in all downloaded FASTQ files was controlled by omitting any read with median phred score less than 30 (roughly 5–10% of the reads were filtered out). Tophat aligned the single-end 75 bp reads to the UCSC hg19 genome model and Cufflinks quantified transcript abundances for the transcripts available in the UCSC hg19 annotations. Cuffnorm (part of Cufflinks) provides gene and isoform normalized abundances in fragments per kilobase of exon per million fragments mapped (FPKM). Since single-end reads were used, Cufflinks could not estimate the fragment length distribution, and instead used an approximate Gaussian distribution with default parameters. Expression levels (in FPKM) at the isoform-level for all samples are summarized in Table 1.

To examine the relative expression levels (transcripts per million, TPM) of Msi2 and Msi2 variant 2 isoforms in normal and cancer cell types we utilized the Cancer RNA-Seq Nexus^57^. The CRN allows genome-wide transcription profiling of cancerous and normal tissues samples. We imposed several restrictions on our analyses, including that the RNA-seq datasets were from the Cancer Genome Atlas (TCGA) and that there were at least 10 samples (cancerous or adjacent normal). Of the 28 cancer types available, 12 fulfilled the criteria and were analyzed here for Msi2 isoform-specific expression, broken down by cancer stage sub-type, where available. Of note, the CRN employs UCSC transcript ID designations and Uniprot protein IDs. The transcript encoding the canonical form of Msi2 (NM_138962 for the above Illumina analyses) corresponds to the UCSC designation uc002iuz and UniProt designation Q96DH6-1. The Msi2 variant 2 transcript (NM_170721) corresponds to the UCSC designation uc002iva and UniProt designation Q96DH6-2. Differential expression between cancerous and normal adjacent tissue datasets were considered significant if the adjusted *p* value was <0.01^57^. Expression data for Breast Invasive Carcinoma, and Endometrial Carcinoma are shown in Table 2, with the other 10 cancer types included in Supplemental Table S1.

### Anchorage-independent colony growth assay

NIH3T3 cells were transfected with constructs expressing the eGFP moiety alone, hMsi2-eGFP, hMsi2 variant2-eGFP, or vBraf using Lipofectamine 2000 (Thermo Fisher Scientific). Approximately 72 hours after transfection the cells were dissociated with trypsin, passed through a single-cell filter, and seeded onto 24-well ultra-low attachment plates. Visualization of eGFP expressing (fluorescent) cells verified equivalent initial transfection efficiency for each construct (using an Olympus IX71 microscope and MetaMorph image analysis software). Cells were cultured for 10 days in DMEM with 0.5% NBCS, with media supplemented every 3 days. After 10 days the numbers of colonies per well were scored. Experiments were performed with 4 replicates per experiment, and repeated on 3 separate occasions.

### Antibodies

Antisera for phosphorylation specific human Msi2 S278 and human Musashi2 S303 were generated by immunizing rabbits with either the peptide CGFPGANS(phospho)PGPVADL or CVGNYISAAS(phospho)PQPGSGF (Proteintech Group Inc.). The antibodies were purified over a peptide-affinity column and used for western blot analyses at 1:1000 dilution. Abcam antibodies to Msi1 were used at 1:1000. Sigma Tubulin antibodies were used at 1:20,000 and Cell Signaling GAPDH antibodies were used at 1:10,000. The phospho-specific MAP kinase antibody (Cell Signaling) was used at 1:1000 to detect activating phosphorylation at Thr202 and Tyr204. The antibody to detect total MAP kinase (Cell Signaling) was used at 1:1000. The GST antibody (Santa Cruz) was used at 1:1000.

### Western blotting

Oocytes were lysed in NP40 lysis buffer^72^ containing sodium vanadate and a protease inhibitor cocktail (Sigma). Where indicated, a portion of the lysate was immediately transferred to STAT-60 for RNA extraction^65^. Gel electrophoresis, transfer and antibody detection was as previously described^45^ except membranes were blocked with 1% bovine serum albumin (Sigma) in TBST for 60 min at room temperature. Experiments were repeated at least twice and representative images are shown.

### Lambda Protein Phosphatase assay

For examination of phosphorylation, oocytes were lysed in a modified NP40 lysis buffer lacking sodium pyrophosphate, sodium vanadate or protease inhibitors and incubated without (mock) or with lambda protein phosphatase (NEB) for 30 minutes at 37 °C. Samples were then analyzed by western blotting.

### Statistical analyses

All quantitated data are presented as mean +/− SEM. Statistical significance was assessed by one-way analysis of variance (ANOVA) followed by the Bonferroni post hoc test or by Student's *t*-test when only two groups were compared. A probability of *p* < 0.05 was adopted for statistical significance.

### Data availability

The datasets generated during and/or analyzed during the current study are available from the corresponding author on reasonable request.

## Electronic supplementary material

Supplementary Data
