# Supplementary material for: Evasion of regulatory phosphorylation by an alternatively spliced isoform of Musashi2
Source: Sci Rep. 2017 Sep 14;7:11503. doi: 10.1038/s41598-017-11917-3 (PMC5599597; doi:10.1038/s41598-017-11917-3)
Supplement: Supplementary file 1 — Supplementary Data [file 41598_2017_11917_MOESM1_ESM.pdf]

Supplementary Data

Evasion of regulatory phosphorylation by an alternatively spliced isoform of Musashi2

Melanie C. MacNicol, Chad E. Cragle, F. Kennedy McDaniel, Linda L. Hardy, Yan Wang, Karthik Arumugam, Yasir Rahmatallah, Galina V. Glazko, Ania Wilczynska, Gwen V. Childs, Daohong Zhou, and Angus M. MacNicol

Supplementary Fig. 3C – lower exposure of pMsi2 images

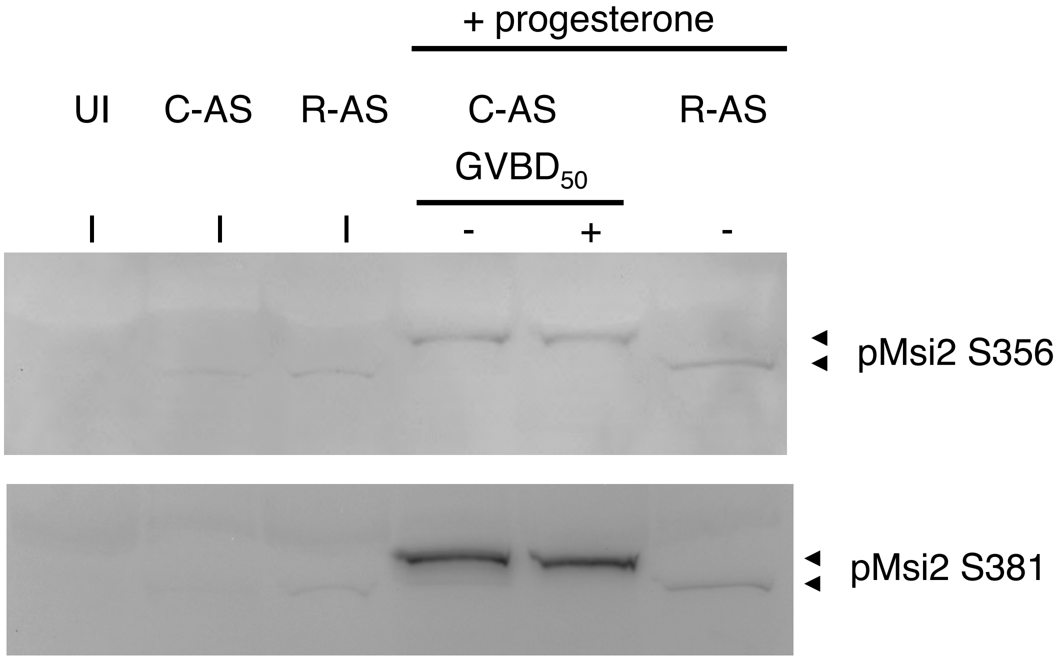

Supplementary Fig. 5F – lower exposure of pMsi2 image

F.

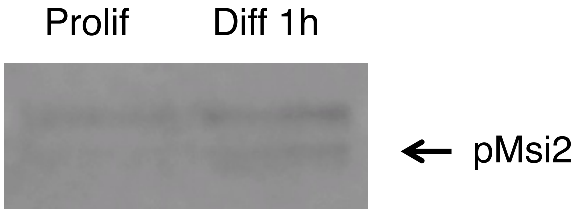

Supplementary Table S1. Differential expression of Msi2 and Msi2 variant 2 transcripts in human cancer vs normal adjacent tissue.

The CRN web portal (http://syslab4.nchu.edu.tw/) was used to interrogate TCGA RNA-seq datasets corresponding to tumor or normal adjacent TCGA RNA-seq datasets for the 10 indicated cancers. The number of total RNA-seq samples are indicated for each condition in parentheses. For each comparison, the RNA-seq expression data were searched for Msi2 expression and the relative expression of the canonical Msi2 (uc002uiz) or Msi2 variant 2 (uc002iva) transcripts determined if the cancerous and adjacent normal data were derived from at least 10 samples as indicated (yes or no). Differential expression of Msi2 or Msi2 variant 2 (Msi2v2) in cancerous tissue vs adjacent normal tissue was not considered significant (ns) if the adjusted *p* value was >0.01. TPM, transcripts per million.

Cancer type	>10 samples?	Msi2			Msi2v2		
		Cancer (TPM)	adjacent normal (TPM)	Adjusted *p* value	Cancer (TPM)	adjacent normal (TPM)	Adjusted *p* value
Colon adenocarcinoma, 479 samples, 10 subsets							
Stage I (74) vs Adjacent normal (40)	Yes	13.35	5.9	3.26E-14	2.38	0.67	9.70E-25
Stage II (29) vs Adjacent normal (40)	Yes	12	5.9	1.57E-04	2.23	0.67	3.09E-11
Stage IIA (137) vs Adjacent normal (40)	Yes	11.99	5.9	1.73E-15	2.67	0.67	2.91E-39
Stage IIB (9) vs Adjacent normal (40)	No						
Stage III (21) vs Adjacent normal (40)	Yes	10.65	5.9	ns	2.35	0.67	3.64E-07
Stage IIIA (12) vs Adjacent normal (40)	Yes	12.03	5.9	4.08E-03	2.74	0.67	4.00E-04
Stage IIIB (56) vs Adjacent normal (40)	Yes	13.71	5.9	9.23E-11	2.73	0.67	2.49E-18
Stage IIIC (39) vs Adjacent normal (40)	Yes	12.63	5.9	7.70E-07	3.46	0.67	4.22E-14
Stage IV (62) vs Adjacent normal (40)	Yes	13.44	5.9	4.38E-14	2.55	0.67	5.93E-21
Lung adenocarcinoma, 572 samples, 9 subsets							
Stage I (5) vs Adjacent normal (59)	No						
Stage IA (133) vs Adjacent normal (59)	Yes	9.65	3.4	1.49E-18	2.79	0.57	2.56E-33
Stage IB (140) vs Adjacent normal (59)	Yes	10	3.4	8.19E-21	2.63	0.57	2.31E-34
Stage IIA (51) vs Adjacent normal (59)	Yes	9.66	3.4	6.27E-09	2.42	0.57	1.66E-15
Stage IIB (73) vs Adjacent normal (59)	Yes	8.95	3.4	6.10E-14	2.21	0.57	2.99E-20
Stage IIIA (73) vs Adjacent normal (59)	Yes	9.13	3.4	1.14E-14	2.42	0.57	1.18E-19
Stage IIIB (11) vs Adjacent normal (59)	Yes	7.81	3.4	ns	2.17	0.57	ns
Stage IV (27) vs Adjacent normal (59)	Yes	9.84	3.4	7.57E-09	2.49	0.57	5.30E-11
Lung Squamous cell carcinoma, 539 samples, 10 subsets							
Stage I (3) Adjacent normal (51)	No						
Stage IA (88) Adjacent normal (51)	Yes	14.89	4.08	3.28E-20	4.86	0.61	5.04E-23
Stage IB (151) Adjacent normal (51)	Yes	15.35	4.08	1.21E-28	4.12	0.61	4.10E-43
Stage IIA (63) Adjacent normal (51)	Yes	18.3	4.08	1.05E-21	5.09	0.61	4.49E-20
Stage IIB (91) Adjacent normal (51)	Yes	15.18	4.08	1.22E-18	3.45	0.61	7.33E-25
Stage III (3) Adjacent normal (51)	No						
Stage IIIA (62) Adjacent normal (51)	Yes	14.71	4.08	6.81E-18	4.41	0.61	4.15E-18
Stage IIIB (20) Adjacent normal (51)	Yes	11.56	4.08	4.19E-06	3.76	0.61	1.25E-06
Head and Neck Squamous cell carcinoma, 486 samples, 6 subsets							
Stage I (27) vs Adjacent normal (43)	Yes	4.65	5.19	ns	1.14	0.86	ns
Stage II (75) vs Adjacent normal (43)	Yes	5.51	5.19	ns	1.58	0.86	8.80E-06
Stage III (76) vs Adjacent normal (43)	Yes	5.86	5.19	ns	1.9	0.86	2.34E-06
Stage IVA (253) vs Adjacent normal (43)	Yes	6.59	5.19	ns	1.97	0.86	2.86E-12
Stage IVB (12) vs Adjacent normal (43)	Yes	6.11	5.19	ns	1.61	0.86	ns
Stomach adenocarcinoma, 431 samples, 12 subsets							
Stage I (3) vs Adjacent normal (35)	No						
Stage IA (14) vs Adjacent normal (35)	Yes	15.76	6.76	2.41E-03	3.5	0.63	6.11E-03
Stage IB (39) vs Adjacent normal (35)	Yes	15.31	6.76	2.83E-05	2.81	0.63	7.73E-08
Stage II (28) vs Adjacent normal (35)	Yes	13.23	6.76	1.48E-05	2.13	0.63	7.37E-08

Stage IIA (36) vs Adjacent normal (35)	Yes	13.07	6.76	1.18E-03	2.1	0.63	4.10E-08
Stage IIB (55) vs Adjacent normal (35)	Yes	15.41	6.76	7.99E-06	2.45	0.63	1.79E-10
Stage III (4) vs Adjacent normal (35)	No						
Stage IIIA (78) vs Adjacent normal (35)	Yes	13.3	6.76	3.14E-06	2.39	0.63	9.04E-18
Stage IIIB (57) vs Adjacent normal (35)	Yes	14	6.76	1.80E-04	2.11	0.63	1.34E-10
Stage IIIC (39) vs Adjacent normal (35)	Yes	15.39	6.76	1.74E-06	1.8	0.63	2.21E-08
Stage IV (43) vs Adjacent normal (35)	Yes	15.21	6.76	5.59E-06	2.26	0.63	3.36E-12
Thyroid carcinoma, 564 samples, 7 subsets							
Stage I (5) vs Adjacent normal (59)	No						
Stage II (53) vs Adjacent normal (59)	Yes	11.89	14.92	ns	2.42	2.48	ns
Stage III (112) vs Adjacent normal (59)	Yes	10.91	14.92	1.62E-03	2.22	2.48	ns
Stage IVA (46) vs Adjacent normal (59)	Yes	10.77	14.92	ns	2.17	2.48	ns
Stage IVB (6) vs Adjacent normal (59)	No						
Esophageal carcinoma, 164 samples, 12 subsets							
Stage I (8) vs Adjacent normal (10)	No						
Stage IB (7) vs Adjacent normal (10)	No						
Stage IIA (39) vs Adjacent normal (10)	Yes	10.76	6.35	ns	2.25	0.57	7.87E-09
Stage IIB (30) vs Adjacent normal (10)	Yes	15.24	6.35	8.90E-03	3.01	0.57	3.59E-07
Stage III (26) vs Adjacent normal (10)	Yes	14.86	6.35	ns	3.26	0.57	2.31E-07
Stage IIIA (13) vs Adjacent normal (10)	Yes	15.86	6.35	ns	2.24	0.57	ns
Stage IIIB (10) vs Adjacent normal (10)	Yes	12.45	6.35	ns	3.57	0.57	3.53E-03
Stage IIIC (7) vs Adjacent normal (10)	No						
Stage IV (5) vs Adjacent normal (10)	No						
Stage IVA (4) vs Adjacent normal (10)	No						
Kidney renal clear cell carcinoma, 601 samples, 5 subsets							
Stage I (265) vs adjacent normal (72)	Yes	11.69	25.78	2.45E-39	1.2	3.59	1.15E-27
Stage II (57) vs adjacent normal (72)	Yes	11.62	25.78	1.54E-11	1.19	3.59	1.72E-20
Stage III (126) vs adjacent normal (72)	Yes	8.21	25.78	3.50E-38	0.92	3.59	8.14E-32
Stage IV (81) vs adjacent normal (72)	Yes	8.08	25.78	5.65E-28	1.03	3.59	6.32E-28
Liver Hepatocellular carcinoma, 391 samples, 9 subsets							
Stage I (167) vs Adjacent normal (42)	Yes	2.16	0.84	1.09E-10	0.73	0.28	9.39E-15
Stage II (84) vs Adjacent normal (42)	Yes	3.19	0.84	3.67E-11	0.89	0.28	4.24E-12
Stage III (3) vs Adjacent normal (42)	No						
Stage IIIA (62) vs Adjacent normal (42)	Yes	3.24	0.84	2.95E-07	1.05	0.28	7.29E-09
Stage IIIB (7) vs Adjacent normal (42)	No						
Stage IIIC (9) vs Adjacent normal (42)	No						
Stage IV (3) vs Adjacent normal (42)	No						
Tumor (14) vs Adjacent normal (42)	Yes	2.62	0.84	8.44E-04	0.99	0.28	4.37E-03
Prostate adenocarcinoma, 549 samples							
Tumor (497) vs adjacent normal (52)	yes	9.9	12.16	4.78E-03	1.88	1.78	ns
